# Giving children a voice: Concept development and foundation of the first Children's council “mental health” in Germany

**DOI:** 10.1002/jcv2.12293

**Published:** 2024-11-25

**Authors:** Christina Bartnick, Hanna Christiansen, Silvia Schneider

**Affiliations:** ^1^ Mental Health Research and Treatment Center (FBZ) Ruhr University Bochum Bochum Germany; ^2^ German Center for Mental Health (DZPG), Partner Site Bochum/Marburg Bochum Germany; ^3^ Department of Child and Adolescent Psychology Philipps University Marburg Marburg Germany

**Keywords:** children, mental health research, participation, patient and public involvement

## Abstract

**Background:**

As summarized by the Lancet Psychiatry Commission on youth mental health (McGorry et al., 2024), the statistics on mental disorders in children are alarming and highlight the need to expand and optimize research on childhood mental health. Although patient and public involvement (PPI) of those affected has the potential to boost both the acceptance and outcomes of research studies, the active involvement of young children, that is, primary school children, in mental health research has been neglected.

**Methods:**

Based on the results of our systematic literature analysis of PPI of children in mental health research, the concept development of the Children's Council ‘Mental Health’ was administered in cooperation with several stakeholders and focus groups (practitioners and researchers in the field of clinical psychology and psychotherapy across the lifespan, representatives from three self‐help organizations for mental disorders, children's focus group). The concept was further optimized in collaboration with the German Children's Fund (Deutsches Kinderhilfswerk e. V.).

**Results:**

The Children's Council was successfully founded with five children aged between 6 and 9 years. Participation is positively received, and each meeting is evaluated using visual measurements. The results from the meetings are presented and discussed. They serve as basis for concrete recommendations for involving young children in mental health research.

**Conclusions:**

To the best of our knowledge, this is the first structured approach involving young children in mental health research studies. The main goal of our study is the direct and immediate empowerment of children, in this case primary school children at the age of 6–12 years. This is associated with a child‐friendly exchange on topics related to mental health, as well as the enhancement of the education process on mental disorders and prevention.

**Key points**

- Mental health research and treatment of children in primary school age is already happening worldwide. However, when it comes to participation in the mental health research process, children have been neglected.
- The main goal of the present study was to give children a voice in mental health research.
- Participation is positively received, all people involved benefit from the collaboration. This might encourage future studies to conduct mental health research involving children.
- The results provide guidelines for the further establishment of participation committees involving young children.
- Adopting an inclusive approach to PPI that incorporates the voices of young children is essential for advancing the field and addressing the multifaceted challenges associated with mental health in this vulnerable population.

## INTRODUCTION

World‐wide, nearly 10 percent of children and adolescents have been diagnosed with various mental disorders (Piao et al., 2022). To date, mental disorders in the age group of 5–14 years have already been ranked second in terms of disability‐adjusted life years (DALYs; Baranne & Falissard, 2018). The findings are consistent with the results of a meta‐analysis that investigated the prevalence of childhood mental disorders in high‐income countries: 5.2% for anxiety, 1.8% for depressive disorder, and 12.7% for any mental health disorder (Barican et al., 2022). These statistics are alarming and highlight the need to expand and optimize research on childhood mental health (McGorry et al., 2024).

While the majority of mental disorders tend to manifest most frequently during the transition from childhood to young adulthood (Colizzi et al., 2020; Kessler et al., 2005; Uhlhaas et al., 2023), the primary emphasis in mental health research continues to be on the adult phase. And even when research is carried out with children and adolescents, it mostly addresses adolescence and not childhood. Despite the fact that the treatment of mental disorders in childhood and youth has shown improvement over the past few decades (e.g., Arnberg & Ost, 2014; In‐Albon & Schneider, 2007; Weisz et al., 2017), there is still a need for further enhancement, as not all children and youth respond equally well to treatment, and relapse rates remain unsatisfactory (In‐Albon & Schneider, 2007; McGorry et al., 2024; Weisz et al., 2017). Furthermore, it appears that existing mental health services are falling short in meeting the needs of affected children and youth. Among the children facing mental health issues, only 44.2% access any form of services, indicating a significant gap in mental health services for children and youth (Barican et al., 2022). In addition, waiting times for psychotherapeutic treatment have nearly doubled since the beginning of the COVID‐19‐pandemic (Plötner et al., 2022). Taken these aspects together, there is an urgent need to transform mental health services so that evidence‐based, effective, and accessible services are being provided (Barican et al., 2022). Expanding the research perspective to the entire lifespan and generating new forms of knowledge are needed for the entire field of mental health research.

A promising approach to be considered appears to be the participation and involvement of those addressed by research. There has been a growing recognition of the importance of engaging diverse stakeholders in the research process, with a particular emphasis on incorporating the perspectives of patients and the wider public. This shift toward patient and public involvement (PPI) can enhance the relevance, quality, and impact of research (Ocloo & Matthews, 2016; Smith et al., 2008). While substantial efforts have been directed toward engaging adults in the research process, the drive for youth patient engagement seems to be progressing at a slower pace (Mawn et al., 2015; McCabe et al., 2023). When it comes to younger children, that is, preschoolers and primary school children, the involvement in mental health research is even more sparse (McCabe et al., 2023).

One of the main reasons why PPI with children has been neglected might be the association of children's participation with several challenges: In addition to fundamental basics, such as age‐specific communication and the way information is presented, there is always a dependency on both the children as well as their caregiver's agreement, consent, the cooperation as well their calendars. This might contribute to worries about needing extra time for preparing and conducting research studies. Furthermore, children bring special challenges, they have shorter attention spans, higher motor activity and impulsiveness, they are more easily frustrated and bored. In addition, children undergo the fastest developmental period in life, and their interests vary rapidly (Handa et al., 2023). All of this results in less adherence in meetings conducted in the participation process (Mawn et al., 2015). Thus, mental health research involving children needs particular guidance. However, given the potential long‐term consequences of mental health issues emerging in early childhood, their and their caregivers inclusion in research endeavors is vital for informing preventive measures and fostering a more inclusive and nuanced approach to mental health promotion and intervention strategies (Jenkins et al., 2020). Mental health research needs to move away from paternalistic approaches toward approaches that also include young children, as the answers that children give in surveys, assessments and interviews are just as important and reliable as those of adolescents and adults (Neuschwander et al., 2013; Silverman & Ollendick, 2005; Weber et al., 2020). Extensive literature demonstrates that parents and children often agree only to a limited extent, or sometimes not at all, when asked about the child's mental disorders (De Los Reyes et al., 2015; Popp et al., 2017). These findings can no longer be ignored, underscoring the necessity of considering children's perspectives. Furthermore, giving children a voice in mental health research processes may benefit directly in several other ways such as skills acquisition (Funk et al., 2012; Gaillard et al., 2018) or strengthening confidence (Holmes et al., 2002), self‐esteem, and knowledge (Brady & Graham, 2019). As previous work by Olsen (2023) has shown, the active participation of children contributes to reducing the sense of powerlessness as well as passive and tokenistic forms of participation; furthermore, participation increases the children's inner strengths and protection forces in the face of unfamiliar and stressful situations. According to previous studies, children want to participate in the decisions that concern them and affect their situation (Van Bijleveld et al., 2015). In fact, children have already demonstrated their ability to do so in city‐planning decisions or local authority decision making (Lundy, 2012), and even when faced with complex family care questions (Van Bijleveld et al., 2015). Taken together, active participation of children in research activities is not only ethically imperative (United Nations, 1989), but also enhances the applicability of findings.

This study adds on existing literature addressing PPI of young people in mental health research, while focusing on the underrepresented age group of primary school children. Although this age group is addressed by research and receives treatment for mental disorders worldwide, their perspectives in mental health research has been ignored so far. To the best of our knowledge, this is the first systematic approach aiming to establish a blueprint for the participation and involvement of children at the age of 6–12 years in mental health research. Furthermore, we aim to present the feedback gathered from the children as transparently as possible, since the lack of reporting this information appears to be the main critical aspect in PPI research (e.g., Todowede et al., 2023; Totzeck et al., 2024). The concept development and foundation of our PPI initiatives with children will be presented, in addition to the main preparational steps needed to provide a basis for further active participation in mental health research studies. Finally, we suggest first guidelines for implementing PPI in mental health research with this age group.

## MATERIALS AND METHODS

### Concept development

The following steps were conducted in order to develop and establish the Advisory Board: At first, a literature search was carried out for mental health research including PPI of children. This systematic literature analysis yielded a lack of studies addressing primary school children (for further results of this study see Totzeck et al., 2024), which is in line with other findings (e.g., McCabe et al., 2023). In a second step, we initiated an exchange of (*n* = 9) practitioners and (*n* = 8) researchers in the field of clinical psychology and psychotherapy across the lifespan to gain more insights into adaptive forms of group sessions with young children. We discussed forming an Advisory Board based on the principles of ‘Young People Advisory Groups’ and discussed possible hurdles and recommendations of ways to conduct PPI with primary school children (i.e., age‐specificity, size of groups, forms of communication). At this point, we decided to form a small group of 5 to a maximum of 8 children, corresponding to a homogeneous age group, with an age range of 5–12 years. The meetings should take place exclusively in person, provide mostly visual information, and one‐fifth of the time should be allocated for playing together. In order to evaluate the meetings, we chose to implement simple visual analogue scales using sad and smiley faces. Two quality rating items were set (‘How comfortable did you feel?’ and ‘How well did you understand everything?’). This initial concept development was evaluated in cooperation with representatives from three self‐help organizations for mental disorders (ADHD, autism spectrum disorder, and anxiety disorders). Here, we also discussed the potential involvement of the children's caregivers, and we decided to provide information about the meetings but to focus solely on the children during the sessions. These concept development ideas were presented to (*n* = 5) children with an age range of 8–11 years. Each child provided their feedback separately. All of them suggested increasing playtime to enhance the enjoyment of meetings and foster a greater sense of comfort among participants. Therefore, the duration of playtime was set to approximately one‐third. Specifications of this concept draft were finalized in cooperation with the German Children's Fund (Deutsches Kinderhilfswerk e. V.). These representatives emphasized the decisions reached in previous group discussions and recommended engaging an external moderator from the Federal Network for Participation to ensure impartial moderation of meetings.

In order to prepare the foundation of the Advisory Board as well as to establish a good collaborative working level, four main research topics were set: (a) implementation of Advisory Boards for primary school children, (b) development of instruments, (c) mental health literacy, and (d) scientific literacy.

### Participants

Children aged between 5 and 12 years were recruited via caregivers at the Mental Health Research and Treatment Center using information flyers about PPI with children in mental health research. This process was adopted to address families who had prior knowledge, experience and/or interest in mental health topics. Further inclusion or exclusion criteria were not set. It was deemed that these children would be ideally placed to comment upon factors, barriers, and recommendations which may be unique to mental health research and practice. Based on the above‐mentioned recommendations, five individuals (*N* = 5; mean age = 6.1 (*SD* = 1.31); age range: 6–9 years; all female) were enrolled in the study, all children as well as their caregivers provided informed consent. All of these children attended every single meeting of the Advisory Board.

### Materials

#### Levels of participation

**FIGURE 1:**
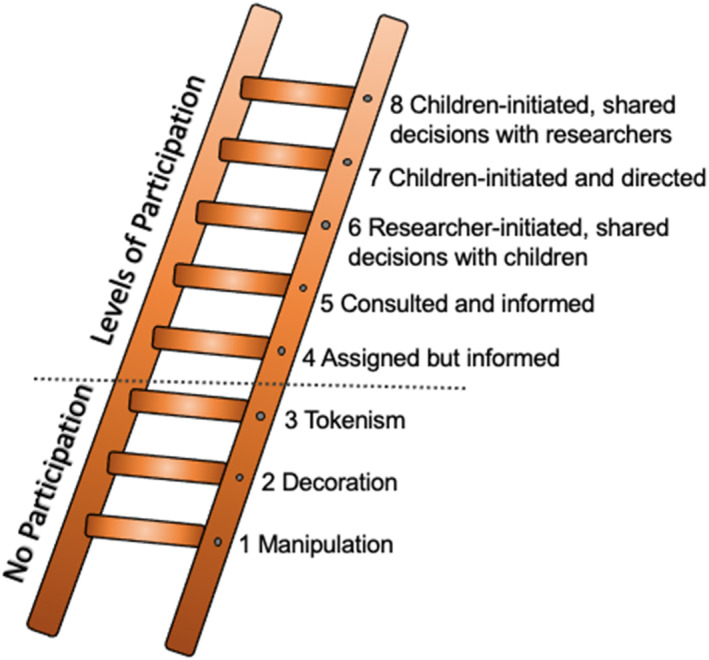
Hart's Ladder of Participation. Note. The Ladder of Participation presenting the levels of children's participation; adapted and modified from Hart (1992).

We adapted Hart's “Ladder of Children's Participation” (1992) to examine the levels of participation together with the participants. Hart's model (1992) includes eight rungs and two main zones (“Non‐Participation” and “Degrees of Participation”). While the three lowest rungs are all designated as “non‐participation”, the top five rungs represent different but valid forms of participation. This resource was used as a basic guide when considering how to include the children in research studies (see Figure 1).

#### Quality ratings

The two quality rating items (see Figure 2A) were presented using 10‐point visual analogue scales, measuring ‘How comfortable did you feel?’ and ‘How well did you understand everything?’. The items were presented on a flipchart and verbally explained. At the end of each meeting, the participants were asked to place adhesive dots on the chart reflecting their responses.

**FIGURE 2a:**
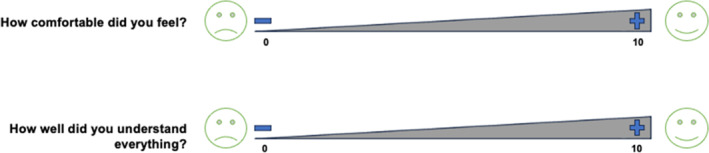
Quality Rating Items. Note. The quality rating items presented were collectively elaborated prior to the first meeting of the Children's Council ‘Mental Health’.

### Procedure

Since its foundation in June 2022, the Children's Council ‘Mental Health’ has met quarterly. Here we present the process and the results from the first five sessions, which served as the foundation and preparational basis for the work of the Children's Council in future mental health research studies. The majority of these meetings (*n* = 3) lasted 90 min; one meeting lasted 120 min, and the initial meeting lasted 180 min. Each meeting was accompanied by either one or two mental health researchers (clinical psychologists and psychotherapists) who presented each research question.

The structure of each session followed the same pattern. After the greeting, there was initially a brief icebreaker game. Subsequently, a review of the previous session was conducted together, and participants were asked what they have retained. Then, two content sequences were carried out, with a break in between for playing activities. Finally, a collective summary was drawn, highlighting what participants particularly enjoyed and what wishes they had for the next meeting. Lastly, evaluation took place. The meetings were moderated and documented by an external moderator (a self‐employed trainer or psychologist not involved in the study) to prevent manipulation of outcomes. After each meeting, each child received a small toy as a token of appreciation for their participation. Due to this setting and the intensive care, no issues with attention, motor restlessness, impulsivity, low frustration tolerance, or similar concerns have arisen so far.

Due to the young age group, the majority of participants did not yet have reading and writing skills. Therefore, the essential information was accompanied by visual instruments. In particular, to enhance structure and participation, a visual agenda (via flipchart) was created for each meeting, supporting interactive transitions between agenda items (templates for visual agendas can be obtained from the authors). Protocols of feedback were administered using paper‐pencil and discussed with the study team after each meeting.

The above‐mentioned instruments were used at the end of each meeting to assess the level of participation (1–8) as well as the quality of meetings. The following four main topics were addressed: (a) implementation of Advisory Boards for primary school children, (b) development of instruments, (c) mental health literacy, and (d) scientific literacy.

Based on the EQUATOR recommendations for reporting PPI in mental health research (see Staniszewska et al., 2017), we used the revised “Guidance for Reporting Involvement of Patients and the Public Checklist—Short‐Form” (GRIPP2‐SF) to report outcomes (see Appendix S1).

## RESULTS

The results of the meetings are clustered according to the main research topics.

### Implementation of Advisory Boards for primary school children

In the initial step, the focus was on establishing a common working level. The previously planned framework was introduced to the children during the first two meetings of the Advisory Board (Hart's level 4). Organizational and communication pathways were discussed, and decisions were made collaboratively. Feedback from the children included preferences regarding the size of the group (5–7 or 8 participants), meeting frequency (originally biannual, but the children preferred quarterly meetings), and meeting duration (optimal duration appeared to be 90 min instead of 120). An overview of the gathered feedback as well as the shared decision results is presented in Table 1. Here we achieved a Hart's participation level of 6.

**TABLE 1:** Overview of the feedback from the children and the shared‐decision consensus.

Topic	Children's feedback	Shared‐decision consensus
Size of groups	‐ *5 participants optimal group size* ‐ *Maximum of 2 or 3 additional participants*	5–8
Frequency of meetings	‐ *Biannual meetings too infrequent, leading to difficulties in recalling information from the last meeting and maintaining continuity in discussions* ‐ *Monthly meetings too frequent due to school, hobbies, family activities, vacations, and holidays* ‐ *No meetings at weekends or during vacations* ‐ *Scheduling meetings every third or fourth month might be optimal*	Quarterly meetings
Duration of meetings	‐ *The optimal duration would be 2 school hours (45 min each) plus a break*.	90 min
Rate of playtime	‐ *Short play at the beginning to warm up (5–10 min)* ‐ *Playtime during break (10–15 min)* ‐ *Short play at the end of the meeting (10 min)*	One‐third of the meeting duration
Participation rate adults to children	‐ *Children should remain in the majority* ‐ *2 adults are sufficient*	A maximum of two adults (i.e., researchers, psychologists) should attend each meeting
Involvement of caregivers	‐ *Advisory Board should focus on the children's perspectives* ‐ *Parents can attend meetings, when specific shared topics are discussed*	Caregivers receive information about relevant results
Minimum age of participants.	‐ *Participants should have sufficient language skills to understand the questions and participate in discussions* ‐ *6 years old is a good age.*	6 years

*Note*: Children's feedback was gathered and summarized; shared‐decision consensus was elaborated in collaboration with children and researchers.

### Development of instruments

Regarding the quality rating of meetings, the children suggested an additional item: ‘How much would you like to come back?’ to reflect their willingness to continue working together. Consequently, they made adjustments to the pictures used to visually represent each of the items (see Figure 2B). These pictures were generated during the meetings using an Artificial Intelligence (AI) program (Microsoft Bing).

**FIGURE 2b:**
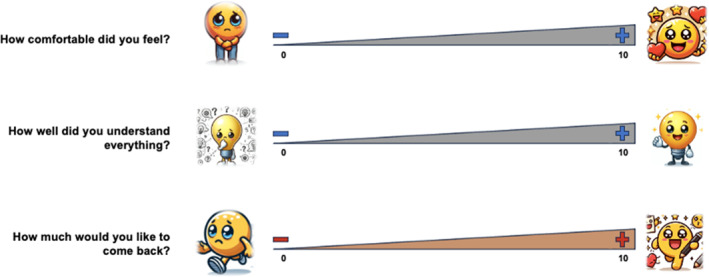
Modified Quality Rating Items. Note. The quality rating items presented were adapted and modified based on the children's feedback. Pictures were jointly developed using an AI program (Microsoft Bing).

**FIGURE 3:**
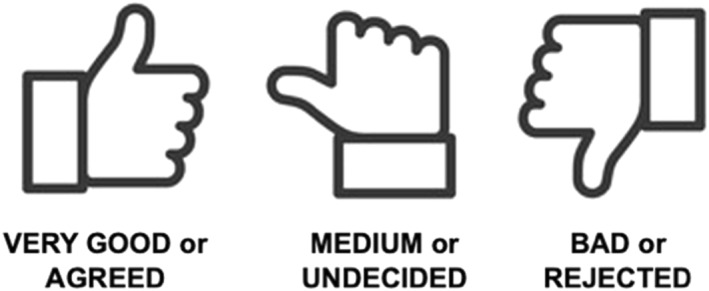
Children's Feedback System Using Thumbs. Note. Illustrated example presenting the simple feedback system “thumbs up” developed by the children.

Furthermore, the children expressed the desire to establish a straightforward feedback system. They proposed a bipolar assessment using their “thumbs up” and “thumbs down,” with a horizontal thumb indicating “average” or “undecided” (see Figure 3). Due to the children's initiative to add an additional item, to elaborate another feedback system, and enhance the visual presentation of questions, a Hart's level of 8 was achieved at this stage.

### Mental health literacy study

The next meeting included an assessment of mental health literacy. This was originally implemented to evaluate the participants' understanding of mental health, but also facilitated the creation of child‐friendly definitions of terms that can be utilized in age‐appropriate settings. The children were presented with questions based on the ‘Mental Health Literacy’ concept by Jorm et al. (1997); Jorm (2000), such as ‘What do you know about mental health? What do you know about mental disorders? What do you know about seeking help?’.

Initially, all individual responses were gathered. Subsequently, during a group discussion facilitated by the external moderator, the children collectively developed child‐friendly definitions for each term (see Table 2). Overall, the children demonstrated a surprisingly comprehensive grasp of mental health and mental disorders. Additionally, knowledge transfer occurred regarding the roles and responsibilities of psychologists, psychotherapists, and psychiatrists. With this approach, we achieved a Hart's participation level of 6.

**TABLE 2:** Mental health literacy items.

Item	Results of the group discussions
**1. What is mental health?**	*Mental health means having a happy and strong mind, just like having a healthy body. It helps us feel good, have fun, play with friends, or learn new things. You can also feel sad, anxious, or upset, but most of the time you feel fine.*
**2. What are mental disorders?**	*Our feelings and thoughts can get a bit mixed up, like a puzzle that needs solving. Mental disorders are when our feelings and minds need extra help to feel better, and that's okay because there are people who can help us put the puzzle pieces back together.*
**3. What do you do, when you feel sad, anxious, worried or upset?**	*I draw, play, or talk about what's bothering me.*
	*I spend time with friends, siblings, parents, or grandparents.*
	*We have worry eaters at home and you can always put a worry in there and then the worry is either very, very small or completely gone.*
	*I cuddle or play with my pets; if you don't have pets, you can cuddle with stuffed animals or play something nice with them.*
	*I always talk to someone, with friends or with mom or dad or with grandma, and not be alone.*
**4. What do you do, when somebody you know experiences a mental disorder?**	*We would go to that person and tell them:*
	*“You look upset. I am here for you.”*
	*“Asking for help is super brave, and there are always people ready to support you.”*
	*“You can talk to me or somebody you trust, a friend, your brother, sister, your parents or a teacher.”*
**5. What does (psycho‐) therapy mean?**	*Therapy is like talking to a special friend who helps you understand and deal with your feelings, thoughts, and problems.*
	*They listen carefully and play fun games or do activities to help you feel better and solve problems.*
	*It's a safe space to talk about anything that's bothering you and learn how to feel happier and more confident.*

*Note*: Jointly elaborated descriptions of mental health literacy terms; child‐friendly definitions.

### Scientific literacy

**FIGURE 4:**
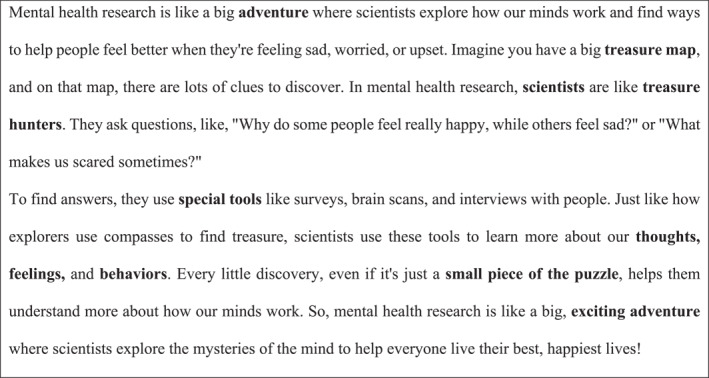
Storytelling Definition of Mental Health Research. Note. The text was jointly elaborated with the children; the bolded words were agreed upon collectively to create the text.

In this last step, scientific literacy was assessed. Similar to the approach used to examine mental health literacy, questions such as, ‘What is research? What do you understand by research on mental health? What is a research study?’ were discussed. At this point, a learning sequence was introduced, during which the meaning of research terms was explored together (e.g., research, study, researcher). Using these terms, we collectively looked for a way to explain mental health research to other children in the same age group in an understandable manner (Hart's participation level 5–6). The children suggested comparing it to a treasure hunt, and together, we developed a story to explain the concept of mental health research in a child‐friendly way (see Figure 4).

#### Evaluation ratings

Overall, participation is positively received. The results of the quality ratings of the meetings are presented in Table 3.

**TABLE 3:** Means and standard deviations for evaluation rates.

Meeting number	How comfortable did you feel?		How well did you understand everything?		How much would you like to come back?	
	*M*	*SD*	*M*	*SD*	*M*	*SD*
1	9.30	0.57	8.04	0.93	9.8	0.39
2	9.64	0.65	7.68	0.54	10.0	0.09
3	9.34	0.40	8.64	1.18	9.72	0.41
4	9.48	0.43	7.78	0.62	9.74	0.48
5	9.52	0.49	8.10	1.17	9.86	0.26

*Note*: Children's evaluations were administered in three modalities in each meeting using visual scales from 0 to 10; *M* = Mean Scores, *SD* = Standard Deviations.

## DISCUSSION

To the best of our knowledge, this is the first structured approach involving young children in mental health research studies. The main goal of our study is the direct and immediate empowerment of children, in this case primary school children at the age of 6–12 years, as they have been left out within PPI in mental health research (McCabe et al., 2023; Totzeck et al., 2024). We aimed to replace the historically paternalistic approach with participation and appreciation of children's voices by the foundation of the first Advisory Board with this age group in mental health research. This has not only the potential to contribute to better outcomes of mental health research studies in childhood, but is also associated with a child‐friendly exchange on topics related to mental health, as well as the enhancement of the education process on mental disorders and prevention.

On the one hand, the concept development of the Children's Council ‘Mental Health’ reflects a holistic approach based on a systematic literature analysis and involving multiple stakeholders. Each‐step of the process contributed to further improvement of the planned framework and enabled the foundation of the probably youngest Advisory Board in mental health research so far. On the other hand, the entire process was characterized by mutual learning from each other. In the end, it was primarily the jointly developed feedback from the children that led to the successful functioning of the Children's Council. Due to the fact that PPI with primary school children had not been conducted in mental health research, there were no guidelines or best practices to be considered or used in the foundation of the Children's Council. During the meetings, the children provided helpful recommendations which led to adaptions of structural and organizational aspects (e.g., meeting duration and frequency), as well as to adjustments in evaluation instruments. Furthermore, the involvement of primary school children in such a young advisory group fostered a collaborative environment conducive knowledge exchange. As highlighted by Jones and Prinz (2005), involving children in decision‐making processes empowers them and promotes a sense of ownership and agency over matters concerning their well‐being. Through this active participation in mental health research, children gained firsthand experience in articulating their perspectives. Moreover, the implementation of this young advisory group serves as a platform for cultivating critical thinking skills and promoting emotional resilience among children. By engaging in discussions on mental health topics and research methodologies, the children were encouraged to think analytically, express themselves confidently, and develop empathy toward others' experiences. These skills not only enhance their capacity to navigate complex social and emotional landscapes, but also equip them with tools for advocating mental health awareness and support within their peer‐groups and communities.

One crucial aspect highlighted by this study is the role of mental health literacy in empowering children as active participants in mental health research. Mental health literacy, defined as “knowledge and beliefs about mental disorders which aid their recognition, management or prevention” (Jorm, 2000, p. 396), serves as a foundational element in equipping children with the necessary knowledge and skills to engage meaningfully in discussions surrounding mental health. By involving the children in the Advisory Board, opportunities arose not only to assess their mental health literacy levels, but also to enhance them through tailored group discussions paired with educational initiatives. All participants enjoyed learning further aspects about mental health. The Children's Council ‘Mental Health’ is currently involved in various research projects and is contributing excellently to improving research. Recently, one member was also a prime example of how dissemination can be collectively promoted by taking the initiative to participate in a radio interview on mental health and presenting the Children's Council. Furthermore, the Children's Council will be the first children's committee to develop a ‘Pixi’ book on the subject of mental health. Pixi books are a series of children's reading and read‐aloud books published in Germany. These books will be made available to children in the future as illustrative learning material on mental health issues.

However, it is essential to acknowledge the challenges associated with implementing such a young advisory group. Firstly, extra time and effort are needed in order to prepare each meeting and discuss protocols afterward. Meetings must be coordinated with both the children's as well as their caregivers' calendars, which also takes additional time. Efforts to mitigate power differentials and ensure inclusivity within the group are paramount to fostering a supportive and respectful environment where all voices are valued and heard. Additionally, children need to be picked up differently than adolescents or adults, which requires additional time for structuring meetings, strengthening motivation or handle bad or tired moods. Considerations regarding safeguarding or special conditions (such as intolerances or allergies) must be carefully addressed to ensure the well‐being and confidentiality of participating children (Smith et al., 2019). Finally, the participation of such a young age group is a lot more complex, as children's narratives are more relational and context dependent. Future research and in particular further PPI initiatives with this age group in mental health research are needed to gain more insights into barriers and facilitators.

The most obvious aspect to criticize is the fact that the Children's Council currently comprises only female participants, lacking representation from a diverse population. Future projects must ensure that more diversity, different social statuses, and other factors are addressed and represented. It would also be worth considering whether specific and tailored patient groups could be conceivable for various research projects. Our approach has already contributed to the establishment of additional children's councils, and precisely because our experience was so positive, we would like to encourage future research studies to also incorporate children's opinions.

### Implications and guideline for practice

Based on the experiences gained from the Children's Council meetings and incorporating direct feedback from the participants, the following guidelines for implementing PPI with primary school children are suggested.

1. Create a trusting, honest, and relaxed atmosphere in small groups of 5 to 8 participants with children being in the majority.
2. Conduct quarterly meetings lasting 90 min, including a playtime of one‐third.
3. Openly inquire about children's preferences (games, drinks, breaks, etc.).
4. For young children, it is especially important to ensure personnel continuity.
5. Avoid written information and use visual instruments and information.
6. Utilize simple methods to gather feedback (i.e., thumbs method).
7. A visual agenda for the flow of each meeting helps provide more structure, preferably with pictures and interactive elements.
8. Report all input made by the children.
9. Include an additional person who either moderates or takes notes during discussions to avoid manipulation of the results.
10. Evaluate the comprehensibility of the content and the well‐being of the children at each meeting.

## CONCLUSION

The globally concerning statistics regarding mental disorders in childhood reflect the need to optimize research and treatment (McGorry et al., 2024). While clinical trials networks in mental health are still limited, those that exist, such as the “Growing Minds Australia Clinical Trials Network” in child and youth mental health (Hawes et al., 2023), are highly relevant. These networks facilitate collaboration and the exchange of experiences to jointly strengthen research and treatment. This is especially true for the inclusion of children and adolescents in the research that addresses them. Involving primary school children in the research process not only serves to deepen the understanding of developmental trajectories, and to democratize science, but also has the potential to positively impact the children's understanding of mental health, well‐being, and the scientific method. Through active participation, children can become co‐creators of knowledge, challenging traditional hierarchies and contributing valuable insights that may otherwise be overlooked. Children can help improve the implementation of mental health research. If we study and treat them, we should also involve them. In conclusion, the foundation and application of a young advisory group with primary school children in mental health research holds immense potential for advancing our understanding of children's mental health needs and experiences. By harnessing the role of mental health literacy and nurturing the knowledge and skills of young children, this approach paves the way for inclusive, participatory research practices that prioritize the voices and well‐being of the next generation.

## AUTHOR CONTRIBUTIONS

**Christina Bartnick**: Conceptualization; Methodology; Project administration; Writing ‐ original draft; Writing ‐ review and editing. **Hanna Christiansen**: Supervision; Writing ‐ review and editing. **Silvia Schneider**: Conceptualization; Methodology; Supervision; Writing ‐ review and editing.

## CONFLICT OF INTEREST STATEMENT

The authors declare that they have no conflicts of interest to disclose.

## ETHICS STATEMENT

Ethics approval was received from the local Ethics Committee of Ruhr University Bochum. The participants' legal guardians, as well as the participants themselves, provided their written informed consent to participate.

## Supporting information

Supporting Information S1

## Data Availability

The authors confirm that the data and material supporting the findings of this study are available within the article and its supplementary materials.
