# Supplementary material for: Giving children a voice: Concept development and foundation of the first Children's council “mental health” in Germany
Source: JCPP Adv. 2024 Nov 25;4(4):e12293. doi: 10.1002/jcv2.12293 (PMC11669770; doi:10.1002/jcv2.12293)
Supplement: Supplementary file 1 — Supporting Information S1 [file JCV2-4-e12293-s001.docx]

**Supporting Information**

**Appendix S1.** *GRIPP2-SF*

| **Section and topic** | **Item** |
| --- | --- |
| **1: Aim** | To develop the first structured approach for PPI with primary school children in mental health research, we aimed to collaboratively conduct, implement, and evaluate the Children’s Council ‘Mental Health’. |
| **2: Methods** | Several stakeholders were involved in the concept development. Nine practitioners and eight researchers in the field of clinical psychology and psychotherapy across the lifespan contributed to group discussions. Additionally, three self-help group organizations for mental disorders and five children (8-11 years) provided their feedback. The final draft was discussed with the German Children’s Fund (Deutsches Kinderhilfswerk e. V.). Five children (6-9 years), two clinical psychology and psychotherapy researchers, and a moderator of the Federal Network for Participation participated in the implementation of the Children’s Council ‘Mental Health’. |
| **3: Study results** | PPI contributed to study outcomes at several stages of the process:  In addition to a structured and holistic approach in the concept development, the direct involvement of children enabled the development of child-friendly instruments to evaluate participation processes, child-friendly definitions of terms regarding mental health literacy as well as scientific literacy. Finally, the children’s feedback on conceptual and organizational structures led to guidelines for future mental health research studies involving primary school children. |
| **4: Discussion and conclusions** | Since PPI with primary school children had not been conducted in mental health research, there were no guidelines or best practices to be considered or used in the conduction of the Children’s Council. During the meetings, the children provided helpful recommendations that led to adaptations of structural and organizational aspects (e.g., meeting duration and frequency). |
| **5: Reflections/ critical perspective** | The key challenge was the prior expectation of how decisions could be made consensually. Initially, it was unclear how well the group would work together and how research expectations could be implemented. However, the implementation worked surprisingly well; the children quickly learned to develop common positions and integrated newly learned information into their feedback. The more fun they had, the greater the outcome of the questions became. |

*Note.* PPI = patient and public involvement.
